# Neuroprotective effects of crude extracts, compounds, and isolated molecules obtained from plants in the central nervous system injuries: a systematic review

**DOI:** 10.3389/fnins.2023.1249685

**Published:** 2023-09-12

**Authors:** Maria Vitoria Nava Moura, Gabriel Mesquita da Conceição Bahia, Marcio Gonçalves Correa, Maíza Amanda Araujo Sarges, Thaís Alves Lobão, Erica Miranda Sanches, Karen R. H. Matos Oliveira, Anderson Manoel Herculano, Carlomagno Pacheco Bahia

**Affiliations:** ^1^Laboratory of Neuroplasticity, Institute of Health Sciences, Federal University of Pará, Belém, Brazil; ^2^Laboratory of Experimental Neuropharmacology, Institute of Biological Sciences, Federal University of Pará, Belém, Brazil

**Keywords:** natural products, neuroprotection, brain Injuries, central nervous system, plants

## Abstract

The number of people with central nervous system (CNS) injuries increases worldwide and only a few therapies are used to mitigate neurological damage. Crude extracts, compounds, and isolated molecules obtained from plants have neuroprotective effects; however, their actions on the central nervous system are still not fully understood. This systematic review investigated the neuroprotective effects of crude extracts, compound, and isolated molecules obtained from plants in different CNS lesions. This PICO (Population/Problem, Intervention, Control, Outcome) systematic review included *in vivo* and *in vitro* studies that used small rodents as experimental models of CNS injuries (P) treated with crude extracts, compounds, and/or isolated molecules obtained from plants (I), compared to non-intervention conditions (C), and that showed a neuroprotective effect (O). Fourteen out of 5,521 studies were selected for qualitative analysis. Several neuroprotective effects (improvement of antioxidant activity, modulation of the inflammatory response, tissue preservation, motor and cognitive recovery) in the brain and spinal cord were reported after treatment with different doses of crude extracts (10 studies), compounds (2 studies), and isolated molecules (2 studies). Crude extracts, compounds, or isolated molecules obtained from plants showed promising neuroprotective effects against several CNS injuries in both the brain and spinal cord, regardless of gender and age, through the modulation of inflammatory activity and oxidative biochemistry, tissue preservation, and recovery of motor and cognitive activity.

## Introduction

Injuries in the central nervous system (CNS) – brain and/or spinal cord – such as traumatic lesions, stroke, and neurodegenerative disease (ND) can compromise motor and/or cognitive functions. Severe injuries may cause permanent disability or even death (Jellinger, 2010). The worldwide number of traumatic brain injuries estimated at 69 million per year (Dewan et al., 2019), strokes 37% and stroke deaths 21% increased significantly between 1990 and 2010 (Bennett et al., 2014). Furthermore, Alzheimer’s disease increased by 147.95% between 1990 and 2019, while Parkinson’s disease increased by 81% since 2000 (Li et al., 2022; World Health Organization, 2022).

Pharmacotherapies with natural products such as crude extracts, compounds, and isolated molecules obtained from plants seem to minimize the injuries caused by damage in the CNS (Pohl and Kong Thoo Lin, 2018). According to Gurib-Fakim (2006), crude extracts are obtained by dissolving dried and separated parts of plants. These extracts can be fractionated to obtain compounds, which in turn can be purified to obtain isolated molecules (Altemimi et al., 2017). It has been recently observed that some crude extracts and compounds can inhibit the nuclear factor kappa B (NF-kB), which prevents the production of inflammatory cytokines and microglial activation in experimental models of some neurological disorders, such as spinal cord injury, multiple sclerosis and amyotrophic lateral sclerosis (ALS; Kyung et al., 2006; Zhu et al., 2006; Sekiya et al., 2009; Chen et al., 2012; Zhang et al., 2015; Lee et al., 2017; Li et al., 2018; Zhou et al., 2018, 2020; Huang et al., 2019; Salehi et al., 2019; Kim et al., 2020; Sharifi-Rad et al., 2020).

Interestingly, evidence suggests that crude extracts, compounds, and isolated molecules obtained from plants can improve the antioxidant system and induce neuroprotective effects in several CNS regions (Kyung et al., 2006; Chen et al., 2012; Badem et al., 2014). Furthermore, antioxidant molecules obtained from plants that interact with the electron transport chain can balance cellular iron levels and improve the antioxidant system (Stoyanovsky et al., 2019; Zhou et al., 2020). All of these actions may converge to induce neuroprotective effects against damages observed in the brain and spinal cord (Chen et al., 2012; Badem et al., 2014; Martinez-Oliveira et al., 2018).

Although some studies indicate potential plant-derived neuroprotective effects, their triggering mechanisms still need to be further elucidated. For instance, Sekiya et al., 2009; Zhou et al., 2018 respectively investigated root extracts of We-Pi-Tang (100 or 200 mg/kg body weight/d up to 16 days of age) and herbs of Huolingshengji Formula (4.5 g/kg.day between 57 to 110 days of age) in an experimental model of amyotrophic lateral sclerosis (ALS). Although both treatments decreased the inflammatory response, only the Huolingshengji Formula induced motor improvement (recovery of upper limb strength and grip movement). Therefore, this study aimed to systematically review the literature on neuroprotective effects induced by crude extracts, compounds, and isolated molecules obtained from plants in experimental models of injured CNS.

## Materials and methods

### Protocol and registration

This study was registered in the Open Science Framework (OFS) database^1^ and reported by following the Preferred Reporting Items for Systematic Reviews and Meta-Analyses (PRISMA) statement (Page et al., 2021).

### Eligibility criteria, information sources, and search

This PICO (Population/Problem, Intervention, Control, Outcome) systematic review included *in vivo* and *in vitro* randomized controlled studies with small rodents as experimental models of CNS injuries (P) treated with crude extracts, compounds, and/or isolated molecules obtained from plants (I), compared to non-intervention conditions (C) and that showed neuroprotective effect (O). English-language studies published between 2002 and July 2023 were searched in PubMed, ScienceDirect, and Scopus databases by two independent authors (M.V.N.M and G.M.C.B) by using the Mesh descriptors ‘animals’, ‘medicinal plants’, ‘fruit’, ‘neuroprotection’, ‘central nervous system’, and ‘spinal cord’. The searches were slightly adapted to each database and followed the PICO inclusion criteria described in the Supplementary material. Search alerts were set to notify the authors of novel publications. Additional primary studies were manually searched in the reference lists of the selected articles.

### Study selection and data collection

The studies were selected by following the criteria: (1) descriptor in the title or abstract, (2) English language, (3) original studies, (4) crude extract, compounds, and/or isolated molecules obtained from specific parts of plants such as roots, whole herb, stem, trunk, flowers, bark/peel, rhizome, and fruit, and (5) *in vivo* and *in vitro* experimental models using small rodents. Reviews, case reports, descriptive studies, opinion articles, technical reports, guidelines, and human trials were excluded.

The Rayyan review assistance tool^2^ was used to exclude duplicates and articles with titles and abstracts that did not meet the eligibility criteria. After full-text reading, the two authors performed the final selection (MM and GM), and disagreements were addressed by a third author (CB). Then, the following data were analyzed: author, study design, number of animals, animal gender and strain, cell line, *in vivo*/*in vitro*, injuries, plant species, part of the plant, extract/molecule, diluent, intervention, evaluation, result, and conclusion.

### Risk of bias In individual studies

The risk of bias for each selected study was independently assessed by the two authors (MM and GM) through the Systematic Review Center for Laboratory Animal Experimentation (SYRCLE), which is based on the Cochrane RoB tool and adjusted to address biases that play a specific role in animal intervention studies (Hooijmans et al., 2014). The following domains were evaluated: allocation sequence, similarity among groups at baseline or confounders adjustment, randomized allocation of experimental and control groups, random housing conditions, blinding of caregivers and researchers, blinding of outcome assessor, incomplete outcome data, selective outcome reporting, and other sources. Domains regarding allocation, housing, baseline similarities, and animal groups assessment are known to influence study outcomes and thus were carefully analyzed to reduce the bias risk and guarantee the quality of the systematic review (Van Zutphen et al., 2001).

In addition, ‘yes’ or ‘no’ questions were answered by the examiners to assess the risk of bias: (1) Is there a possibility that the results are biased? (2) Do the results have factors that confuse the interpretation of the results? (3) Could the study results occur by chance? Articles that received mostly ‘no’ answers were considered methodologically viable and with low bias risk.

## Results

### Study selection

**Figure 1:**
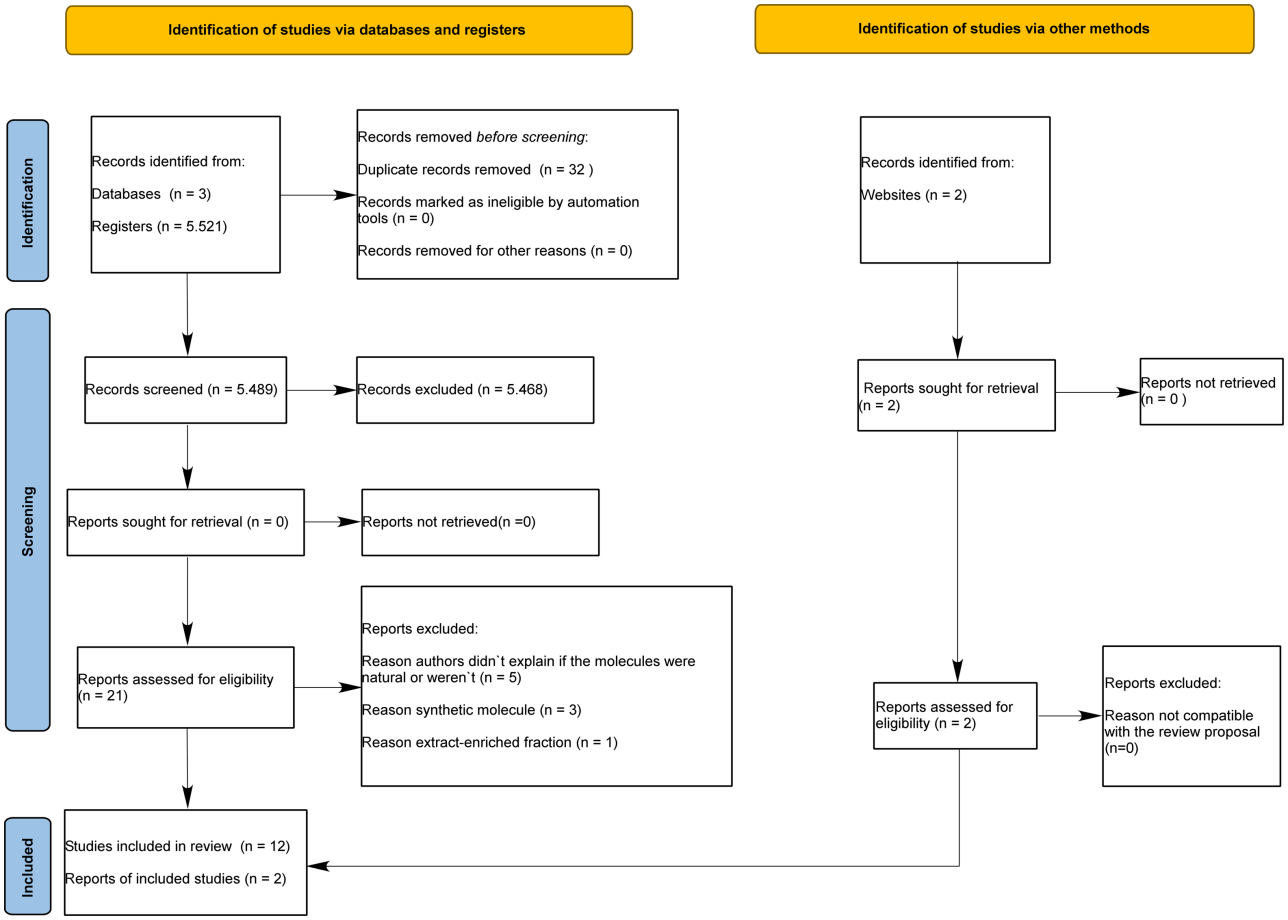
The PRISMA flowchart.

Among 5,521 articles, 32 were duplicates and 5,489 were excluded during title and abstract reading. Thus, 21 articles were fully read and 2 articles were found and added by other methods. Next, 5 articles were excluded since the origin of the molecules (natural or synthetic) was not described, 3 articles were excluded due to the use of synthetic molecules, and 1 article was excluded due to the use of an extract-enriched fraction. Finally, 14 articles were eligible for the qualitative synthesis (Figure 1).

### Study characteristics

Ten studies evaluated the neuroprotective effects induced by crude extracts obtained from plants (Sekiya et al., 2009; Guimarães-Santos et al., 2012; Badem et al., 2014; Zhang et al., 2015; Lee et al., 2017; Li et al., 2018; Martinez-Oliveira et al., 2018; Zhou et al., 2018; Huang et al., 2019; Kim et al., 2020), 2 studies used isolated molecules (Kyung et al., 2006; Zhu et al., 2006), and 2 studies reported the effect of a compound (Chen et al., 2012; Zhou et al., 2020).

The crude extracts, compounds, and/or isolated molecules were extracted from herbs in 4 studies, roots in 4 studies, leaves in 2 studies, rhizomes in 2 studies, fruit in 2 studies, flower in 1 study, tree in 1 study, and trunk in 1 study. Among the experimental models, 4 studies induced spinal cord injury (Kyung et al., 2006; Chen et al., 2012; Zhang et al., 2015; Zhou et al., 2020), 5 studies analyzed multiple sclerosis (Zhu et al., 2006; Lee et al., 2017; Li et al., 2018; Huang et al., 2019; Kim et al., 2020), 2 studies evaluated amyotrophic lateral sclerosis (Sekiya et al., 2009; Zhou et al., 2018), 1 study induced spinal cord ischemia (Badem et al., 2014), 1 study reproduced acute damage to the motor cortex (Guimarães-Santos et al., 2012), and 1 study replicated Alzheimer-like disease damage in the hippocampus (Martinez-Oliveira et al., 2018). Ten *in vivo* studies, 1 *in vitro* study, and 3 both *in vivo* and *in vitro* studies were selected. Six studies used ethanol as a diluent, two studies used distilled water, one study was not possible to determine, and the other five studies used saline, DMSO, methanol, Tween, or alcohol.

### Risk of bias

**Figure 2:**
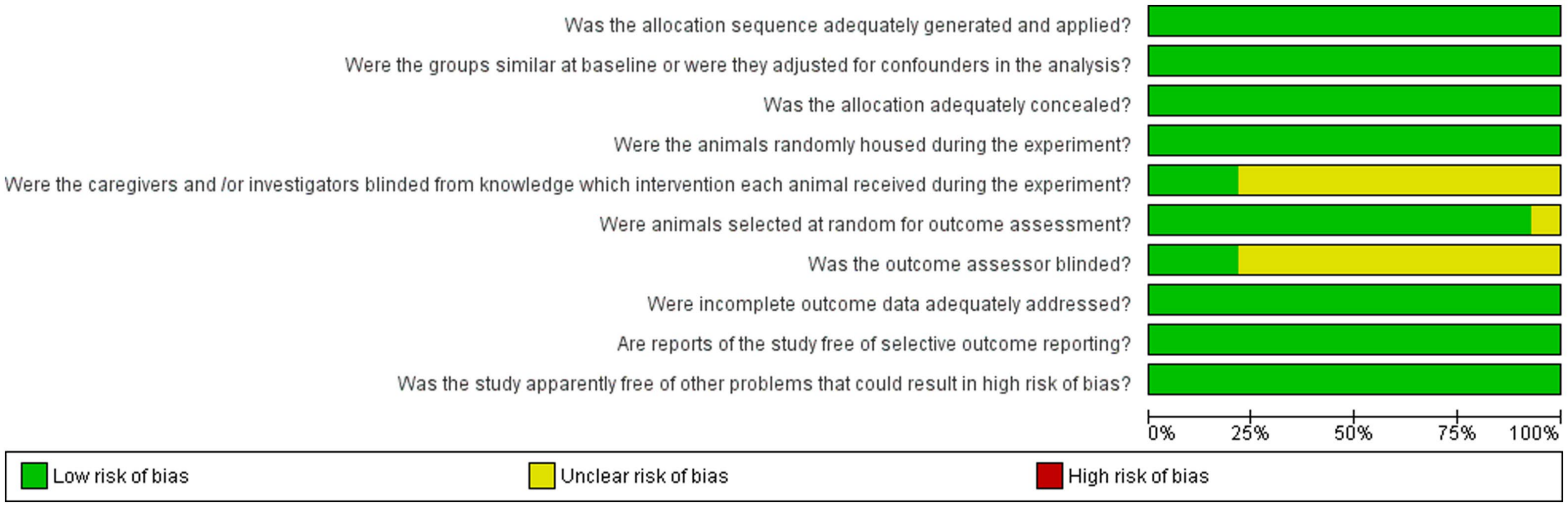
Risk of bias for 10 domains of the studies qualitatively analyzed. Green and yellow colors indicate low and unclear risk of bias, respectively.

**Figure 3:**
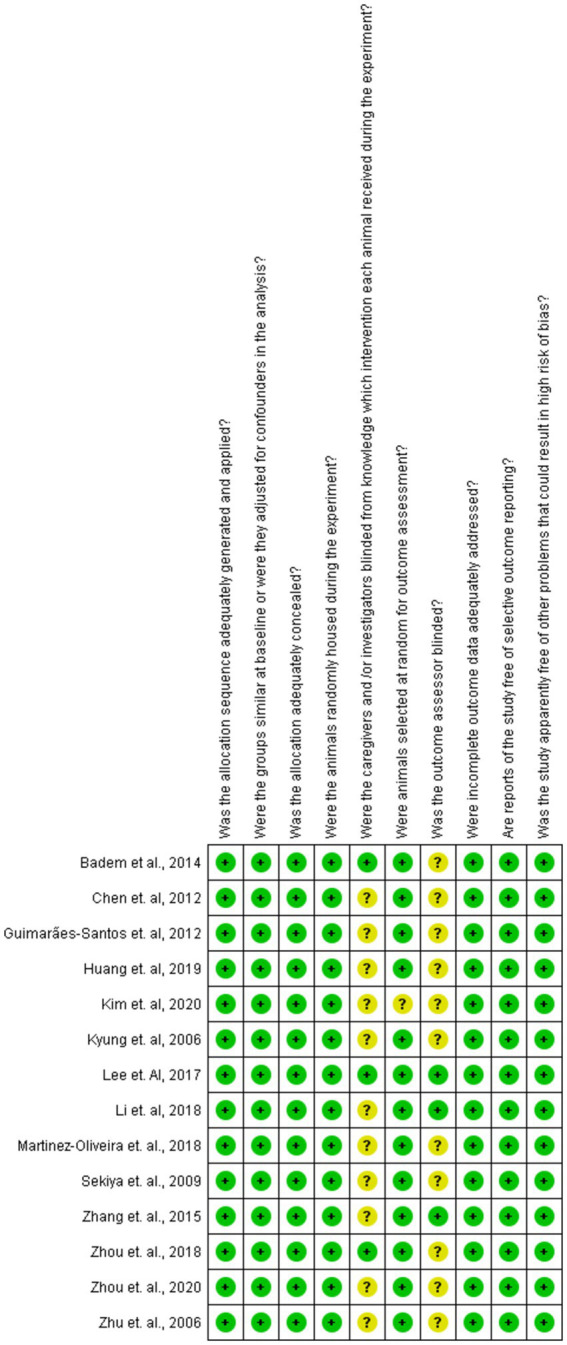
Percentage of risk of bias levels for each domain.

The detailed analysis of the 10 main domains indicated a low risk of bias regarding allocation sequence since most items were classified as ‘yes’. No study was found with a high risk of bias. The ‘random housing conditions’, ‘incomplete outcome data’, and ‘other sources’ domains were found with low risk, while the ‘blinding of caregivers and researchers’ and ‘blinding of outcome assessor’ domains were classified as unclear in most of the studies (Figures 2, 3).

### Results of individual studies and summary

The selected studies evaluated a total of 28 plant species (Table 1). *Ginkgo biloba, Radix rehmanniae Preparata,* and *Zingiber officinale* were reported in 2 studies each. Other species (*Acanthopanax senticosus Harms*, *Copaifera reticulata* Ducke, *Alpinia oxyphylla Miq.*, *Vitis vinifera L.*, *Vigna angularis*, *Coptis chinensis FRANCH*, *Scutellaria baicalensis GEORGI*, *Smallanthus sonchifolius*, *Rheum officinale* BAILLON, *Panax ginseng* C. A. MEYER, *Aconitum japonicum* THUNBERG, *Glycyrrhiza glaba* LINN, *Astragalus membranaceus*, *Angelica sinensis*, *Paeonia lactiflora* Pall, *Ligusticum chuanxiong*, *Prunus persica*, *Carthamus tinctorius* L, *Pheretima aspergillum*, *Epimedium herb*, *Radix astragali*, *Fructus corni, Poria cocos*, and *Atractylodes macrocephala Koidz*, *Periploca sepium* Bge) were reported in 10 studies.

**Table 1:** Data retrieved from the selected articles.

Author	Study design	Animals number	Animal gender and strain	Cells line	*In vivo*/*In vitro* experiments	ND	Plant species	Part of the plant	Extract/molecule	Diluent	Intervention	Evaluation	Result	Conclusion
Badem et al. (2014)	Animal’s model	24 (3 groups of 8): group 1 (sham); group 2 (IR) and group 3 (IR + EGb)	3 to 4-month-old healthy *Spraguee Dawley* rats (250 to 350 g body weight)	Not applicable	*In vivo*	Spinal cord ischemia	*Ginkgo biloba* tree	Leaves	Extract	Alcoholic	Treatment group: 100 mg/Kg/day orogastric dose 3 days pre-surgery and 2 days post-surgery	Neurological outcome (neurological performance scores); MDA; SOD, GSH and GSH-Px; spinal cord tissue preservation (H&E);	θ SOD θ GSH-Px θ MDA ↓ GSH	Although both oxidative stress and lipid peroxidation analyses did not show a clear effect, histopathological analysis showed an improvement in the treated group.
Chen et al. (2012)	Animal’s model	Spinal cord samples obtained from 5 embryos and cultivated into five 24-well plates	15-day pregnant *Spraguee Dawley* rats	Not applicable	*In vitro*	Spinal cord injury	*Acanthopanax senticosus Harms and Ginkgo biloba*	Herb and tree, respectively	*Acanthopanax senticosus* saponins (ASS) and ginkgolides	Methanolic	Gin group: spinal cord samples pre-incubated with 800 μl of 37.5 μg/ml Gin 24-h after culture and 24-h before hypoxia injury; ASS group: spinal cord samples pre-incubated with 800 μl of 50 μg/ml Gin 24-h after culture and 24-h before hypoxia injury;	Neuron survival rate (MTT assay); LDH release; detection of HIF-1α protein expression (Western Blot); morphological observation;	↑Fibroblasts, neuroglial cells and neurons number ↑survival rate ↓LDH release ↑HIF-1α expression	Treatment with Gin and ASS increased the number of fibroblasts, neuroglia cells and neurons, enhanced the viability of spinal cord cells by decreasing LDH release and promoted neuroprotection under hypoxia through the modulation of HIF-1α expression
Guimarães-Santos et al. (2012)	Animal’s model	21 animals (3 groups of 8): control; group 1 (400 mg/Kg); group 2 (200 mg/Kg).	Male adult *Wistar* rats	Not applicable	*In vivo*	Excitotoxic injury in the motor cortex	*Copaifera reticulata* Ducke	Trunk	Extract	5% Tween + sterile saline	Group 1: single dose after surgery Group 2: two daily doses during 3 days	Motor cortex tissue preservation (H&E staining); necrosis cell death; the presence of polymorphonuclear and mononuclear cells (cresyl violet);	↓ Tissue necrosis ↓ cellular infiltration ↓ microglial activation↓ neutrophil activation	COR treatment induced tissue preservation and decreased the recruitment of neutrophils and microglial activation in the injury site compared to vehicle animals
Huang et al. (2019)	Animal’s model	Vehicle; Group 1: 300 mg/kg of Alpinia oxyphylla (AO); Group2: 1,000 mg/kg of AO	8-week-old female C57BL/6 mice	Splenocyte ELA cells	*In vitro and in vivo*	Multiple sclerosis	*Alpinia oxyphylla Miq.*	Fruit	Extract	Ethanolic	Oral or intraperitoneal administration of AO-1 (300 and 1,000 mg/kg), 7 days after injury, during 14 days	Constitution analysis of AO-1; Total proteins (Western Blot); cell survival rate (MTT assay); IL-7 detection from ELA cells genes expression from RNA (total PCR); histological analysis	↓ score of experimental autoimmune encephalomyelitis (EAE) ↓demyelination ↓inflammation ↓infiltration of immune cells (CD4^+^ T cells, CD8^+^ T cells, and CD11b^+^) ↓Th1/Th17 response	AO-1 treatment ameliorated the severity of EAE (score reduction). The regulation of Th1/Th17 response reduced the infiltration of others immune cells, which reduced inflammation and demyelination, and lead to tissue preservation
Kim et al. (2020)	Animal’s model	Normal group; EAE-vehicle; EAE + OAA (10 mg/kg); EAE + OAA (30 mg/kg)	7 to 8-week-old female C57BL/6 mice	Not applicable	*In vitro and in vivo*	Multiple sclerosis	*Vigna angularis*	Herb	Extract	Ethanolic	Group 1: 10 mg/kg of OAA dissolved in 1% (w/v) carboxymethylcellulose, from days 11 to 21 post-immunization Group 2: 30 mg/kg of OAA dissolved in 1% (w/v) carboxymethylcellulose, from days 11 to 21 post-immunization	T cell proliferation assay Cytokines level detection; total RNA content (total PCR); proteins measure (Western Blot); H&E staining; antibodies detection (immunohistochemistry)	↓Hind limb paralysis ↓T cell proliferation ↓Proinflammatory cytokines ↓Macrophages ↓TRL2 level ↓Myd88 ↓IRAK4 ↓TRAF6	OAA treatment showed therapeutic properties, mainly through TRL2 signaling pathway, which inhibited the activation of proinflammatory proteins, reduced T cell proliferation, and improved hindlimb function
Kyung et al. (2006)	Animal’s model	160 animals allocated in 3 SCI groups of 40 animals,1 treated group of 30 animals, and control groups of 10 animals	5-week-old adult female *Wistar* rats (250 g)	Not applicable	*In vivo*	Spinal cord injury	*Zingiber officinale L.*	Ground rhizomes	Shogaol	DMSO (dimethylsulfoxide) + matrigel	Treatment group received different doses of shogaol dissolved in dimethylsulfoxide and matrigel with different concentrations into injured site (0, 20, 50 and 100 lg/kg)	Spinal cord tissue preservation; identification of astrogliosis; hypomyelination and neuronal cell death; oxidative stress signaling; functional recovery;	↓ Apoptosis ↑ ED-1 cells ↓ leukemic B cells ↑ Bcl2 lymphomas ↓ astroglia cells ↑ Function improvement ↑ signal transducer and activation of transcription-3 ↑ Jak2 ↓ p-STAT, p-SAPK /JNK, P38, p-ERK1 / 2, EGFR	Shogaol administration attenuated apoptosis cell death and reduced astrogliosis and hypomyelination. In comparison to control, shogaol-treated rats recovered hindlimb reflexes more rapidly and a higher percentage regained response.
Lee et al. (2017)	Animal’s model	24 animal (3 groups, n = 6 per group): vehicle; EAE; EAE + Samhwangsasim-tang extract (SHSST) group + SHSST alone group	Female adult C57BL/6 mice	Not applicable	*In vivo*	Multiple sclerosis	*Coptis chinensis FRANCH and Scutellaria baicalensis GEORGI*	Rhizomes (*C. chinensis* and *R. officinale*) + roots (*S. baicalensis*)	Extract	Ethanolic	EAE-SHSST group: [200 mg of MOG35-55, s.c. C 300 or 600 mg/kg of SHSST, p.o.] SHSST alone group: [vehicle treatment, s.c. C 600 mg/kg of SHSST, p.o.]	Identification of SHSST extract (qualitative HPLC analysis); immunohistochemistry; gene expression from RNA (RT-PCR); total proteins (Western Blot); cells quantification (flow cytometry)	↓ EAE score ↓ cellular infiltration ↓ demyelination ↓ infiltration of myeloid cells ↑ blood–brain barrier integrity ↓ CD3 cells ↓CD4^+^ T cells θ CD8^+^ T cells ↓iNOS ↓ COX-2 ↓ NF-kB ↓ MAPKs ↑ body weight	SHSST extract can attenuate EAE by suppressing TH1 cells, mRNA expression and proteins associated with oxidative stress and inflammatory response. The treatment also down-regulated nuclear factor-kappa B and mitogen-activated protein kinases signal pathways in the spinal cords; thus, the animals gained body weight
Li et al. (2018)	Animal’s experiment and human cell lines	14 animals (2 groups of 7): vehicle and RR treatment	Female C57BL/6 N mice (8–10 week old)	SH-SY5Y cells	*In vivo* and *in vitro*	Multiple sclerosis	*Radix Rehmanniae Preparata*	Herb	Extract	Ethanolic	Treatment group: oral administration of 3.7 g/kg/day of RR extract dissolved into 0.3% of sodium carboxymethyl cellulose	Inflammation and demyelination (H&E e LFB staining); infiltration of T-cells, macrophages, and microglia; ONOO-scavenged by RR extract;	↓ Cellular infiltration ↓ demyelination ↓ CD3^+^ T, and CD11b^+^ CD45 ↓ NF-κB signaling ↑ ONOO- scavenging	RR treatment effectively ameliorated clinical disease severity, inhibited inflammation/demyelination in spinal cord, and alleviated CNS infiltration of encephalitogenic T cells and activated macrophages. Furthermore, RR treatment suppressed the NF-κB signaling pathway in the splenocytes of EAE mice. The *in vitro* experiments on macrophages and neuronal cells exerted consistent results with the *in vivo* animal experiments
Martinez-Oliveira et al. (2018)	Animal’s model	48 animals (6 groups of 8): control; YR (40 mg/Kg); YL (40 mg/Kg); Aβ; YR (40 mg/Kg) + Aβ; YL (40 mg/Kg) + Aβ	60 days-old male *Wistar* rats	Not applicable	*In vivo*	Alzheimer’s disease	Yacon [*Smallanthus sonchifolius* (Poepp. and Endl.) H. Robinson]	Leaf and roots	Extract	Ethanolic	Group 1: oral administration of 40 mg/kg of Yacon root extract + surgery for intrahippocampal injection of Aβ; Group 2: oral administration of 40 mg/kg + surgery for intrahippocampal injection of Aβ. Both groups were daily treated for 14 days.	Total cholesterol and fractions, triglycerides, glucose, liver function marker enzymes, kidney function (urea, creatinine, and uric acid); oxidative stress and lipid peroxidation; SOD, CAT, GSH, and GPx; memory and behavior test;	↑ CAT ↑SOD ↑ GSH ↑ GSH-Px ↑ behavior and cognition	Treatment with Yacon leaf and root extracts improved oxidative stress parameters in the hippocampus. Yacon leaf extract demonstrated better neuroprotective effects than root extract and also protected against memory deficits related to Aβ-protein-induced neurotoxicity
Sekiya et al. (2009)	Animal’s model	50 animals (5 groups of 10): W100, W200, vehicle, wild, and Riluzole	6-week-old mice carrying a mutated human SOD1 gene and wild-type littermates	Not applicable	*In vivo*	Amyotrophic lateral sclerosis (ALS)	*Rheum officinale* BAILLON, *Panax ginseng* C. A. MEYER, *Aconitum japonicum* THUNBERG, *Zingiber officinale ROSCOE*, *Glycyrrhiza glaba* LINN. var. glandulifera REGEL et HERDER	Roots	Extract	Distilled water	W100: 100 mg/kg body weight/d W200: 200 mg/kg body weight/d. In both groups, the animals were treated from 7 to 16 weeks of age	Motor function (Rota-Rod Test) hind limb strength (Wire Hang Test) grip strength test motor neuron umber (histological analysis) glial fibrillary acidic protein detection (immunohistochemistry) expression levels of GFAP, HO-1, and iNOS (immunohistochemistry) total protein content (Western Blot)	θ Body weight θ hind limb strength θ grip strength test ↑ motor function θ disease onset at W100 group ↓disease onset at W200 group ↓ neuronal loss ↓ astrocyte ↓ microglial cells ↓HO-1 at W200 group ↓ iNOS at W200 group ↑ nNOS	Although body weight, hindlimb strength and grip strength parameters were not significantly improved, Wen-Pi-Tang extract delayed the ALS disease onset, by inhibiting astrocytic and microglial proliferation and reduced neuronal loss
Zhang et al. (2015)	Animal’s model	78 animals allocated into: sham-operated control (6 rats), SCI (18 rats); Buyang Huanwu decoction [BYHWD (18 rats)], neuron stem cells [NSCs transplantation (18 rats)] and NSCs transplantation combined with BYHWD (BYHWD + NSCs; 18 rats)	Female *Sprague–Dawley* rats (weight, 220–250 g)	SD embryonic neural tube (11.5 days)	*In vivo*	Spinal cord injury	*Astragalus membranaceus* (Fisch.) Bge. var. *mongholicus* (Bge.) Hsiao; *Angelica sinensis* (Oliv.) Diels; *Paeonia lactiflora* Pall; *Ligusticum chuanxiong*; *Prunus persica* (L.) Batsch; *Carthamus tinctorius* L; *Pheretima aspergillum*	Root: *A. membranaceus*; *A. sinensis*; *P. lactiflora*; *L. chuanxiong* Ripe seed: *P. persica* Flower: *C. tinctorius; P. aspergillum*	Extract	Could not identify	BYHWD group: 18 rats BYHWD + NSCs: 18 rats. Both groups received 14.8 g/kg/day of BYHWD, through intragastric infusion once a day. Evaluations were performed at 7, 14 and 28 days after treatment	Motor function (BBB locomotor rating scale) neural stem cells detection (immunofluorescence staining) oligodendrocytes differentiation (immunofluorescence staining) astrogliosis activity (immunofluorescence staining) cell counting of NSCs	↑ BBB score ↑ myelination ↑motor function ↓ astrogliosis	The administration of BYHWD reduced the astrocytes activity, increased myelination, and improved neurological and motor functions
Zhou et al. (2018)	Animal’s model	18 animals (3 groups of 6): vehicle, M-HLSJ-treated and riluzole-treated	Transgenic male mice expressing mutant human SOD1G93A	Not applicable	*In vivo*	Amyotrophic lateral sclerosis (ALS)	*Epimedium Herb, Radix Astragali, Fructus Corni, Radix Rehmanniae, Poria cocos and Atractylodes macrocephala Koidz*	Herb	HLSJ	Distilled water	M-HLSJ treated group received 4.5 g/kg·d HLSJ suspended in ddH_2_O, from the disease onset until mice death	Assessment of disease onset (Rota-Rod Test); grip strength of hind limbs; survival of motor neurons (Nissl staining and TUNEL); expression of apoptosis-related protein (Western Blot); activation of microglia and astrocytes (anti-Iba-1 and anti-GFAP immunostaining) pathological analysis of gastrocnemius muscles	↑ Lifespan ↓MDA ↓ muscle atrophy ↑ motor neuron survival ↓ Bax ↓ cleaved-caspase-3 ↓ Cyt c ↑ Bcl 2 ↑ grip strength of hind limbs ↓ gastrocnemius muscle degeneration ↑ neuromuscular junctions ↓ NADH ↓ glial cells ↓ TNF-α, Cox2 and iNOS	HLSJ prolonged lifespan, extended disease duration, prevented motor neuron loss, alleviated the atrophy of gastrocnemius muscle, and reduced apoptosis and inflammation in the spinal cord
Zhou et al. (2020)	Animal’s model	16 animals allocated in 4 groups: sham, SCI, PACs5 (5 mg/Kg), PACs10 (10 mg/Kg)	10 to 12-week-old female C57BL/6 mice (24 to 26 g body weight)	Not applicable	*In vivo*	Spinal cord injury	*Vitis vinifera L.*	Fruit	Proanthocyanidin	0.9% saline solution	PACs5 and PACs10: intraperitoneal administration between the fifth and tenth day after injury	Basso mouse scale and footprint test, histological and immunohistochemical staining, determination of protein	↓ TBARS ↓ ACSL4 ↓ Alox 15B ↑ GSH ↑ GPX4 ↑ locomotive function	Traumatic injury increased iron levels; however, treatment with PACs 5 and 10 decreased iron levels and restored locomotive function. PACs treatment may be a potent antioxidant drug that promotes SCI locomotive repair
Zhu et al. (2006)	Animal’s model	30 animals (2 groups of 15): control (1.6% ethanol + PBS) and PSE (10 mg/kg/day)	6 to 8-week female C57BL/6 mice	Not applicable	*In vivo*	Multiple sclerosis	*Periploca sepium* Bge	Stem bark	Periplocoside E (PSE)	Ethanolic	PSE group: 10 mg/kg/day daily injected for 16 days	IFN-γ levels measurement (cytokine assay) CXCR3 and CCR5 mRNA expression assay genes expression (RT-PCR) histopathological analysis	↓ myelin oligodendrocyte glycoprotein (MOG)-specific immune response ↓ demyelination ↓ inflammation ↓CCR5 ↓CXCR3 ↓ IL-12 ↓ IFN-γ ↓ primary T cell infiltration ↓ chemokine (CCL3, CXCL9, and CXCL10) ↓ macrophages ↓ CD4 ↓ CD8 ↓ CD11b	PSE administration reduced encephalomyelitis allergy symptoms by suppressing inflammatory cytokines, T cells infiltration, and expression of genes that trigger multiple sclerosis

↑ = improvement; ↓ = decrease; θ = no change.

Regarding the experimental model of CNS injuries, 5 studies reproduced multiple sclerosis (Kyung et al., 2006; Zhang et al., 2015; Lee et al., 2017; Li et al., 2018; Huang et al., 2019; Kim et al., 2020), 4 studies induced spinal cord injury (Kyung et al., 2006; Chen et al., 2012; Zhang et al., 2015; Zhou et al., 2020), 2 studies reproduced amyotrophic lateral sclerosis (Sekiya et al., 2009; Zhou et al., 2018) and 3 studies evaluated spinal cord ischemia, excitotoxicity into the motor cortex, and Alzheimer’s disease (Guimarães-Santos et al., 2012; Badem et al., 2014; Martinez-Oliveira et al., 2018).

Four studies used two plant parts: herb and the tree (Chen et al., 2012); rhizome and the root (Lee et al., 2017); root and the flower (Zhang et al., 2015); leaf and the root (Martinez-Oliveira et al., 2018). One study used only the root (Sekiya et al., 2009), 3 studies used only herbs (Li et al., 2018; Zhou et al., 2018; Kim et al., 2020), 2 studies used only the fruits (Huang et al., 2019; Zhou et al., 2020), 1 used only leaves (Badem et al., 2014), 1 used only rhizome (Kyung et al., 2006), 1 study used only the stem bark (Zhu et al., 2006), and 1 study used only the trunk (Guimarães-Santos et al., 2012).

The orogastric administration of *Ginkgo biloba* tree extracts at 100 mg/Kg/day for 3 days before and 2 days after surgery reduced lipid peroxidation by means of GSH and MDA decrease; in addition, the histopathological analysis revealed less pronounced damage to the spinal cord (Badem et al., 2014). The oral administration of a single 400 mg/kg dose or two 200 mg/kg doses of *Copaifera reticulata* oil-resin decreased the necrosis area in the motor cortex (M1) of adult rats, reduced the number of mononuclear and polymorphonuclear cells; in addition, the neutrophilic infiltration and microglial activation were reduced to 39 and 62%, respectively (Guimarães-Santos et al., 2012).

The *Alpinia oxyphylla Miq.* fruit extract was orally or intraperitoneally administered (300 or 1,000 mg/kg) 7 days after spinal cord injury and for 14 days. This treatment reduced the severity of encephalomyelitis and decreased demyelination and inflammation in the injured area. Furthermore, the extract inhibited the expression of T-bet e RORγt proteins into the spleen and thus reduced the activity of CD4^+^, CD8^+^, and CD11^+^ cells (Huang et al., 2019).

*Vigna angularis* herb crude extract was orally administered at 10 and 30 mg/kg doses between 11 to 21 days after immunization and attenuated the symptoms of multiple sclerosis. The treatment mitigated hind limb paralysis, suppressed specific T cell proliferation, reduced mRNA expression of pro-inflammatory cytokines (TNF-a, IL-1b, and IL-6), and macrophage accumulation in the CNS, and inhibited the TRL2 signaling pathway (Kim et al., 2020).

The crude extract of Radix rehmanniae (RR) herb was orally administered at 3.7 g/kg/day and the ONOO− scavenging capability was evaluated in SH-SY5Y cells. The treatment attenuated the spinal cord inflammation, demyelination process, and oxidative stress. Flow cytometry analysis showed reduced levels of CD3 + T cells and CD11b + CD45 macrophages in the brain and inhibition of NF-κB signaling (Li et al., 2018).

The ethanolic crude extract of Samhwangsasim-tang formula was prepared to treat multiple sclerosis at 300 or 600 mg/kg post-immunization. The treatment inhibited body weight loss, reduced the infiltration of the immune and myeloid cells into the spinal cord, and preserved the integrity of the blood–brain barrier (Lee et al., 2017).

Crude extracts of *Smallanthus sonchifolius* leaves and roots were orally administered at 40 mg/kg for 14 days to treat Alzheimer’s disease. The treatment improved memory, reduced glucose levels, lipid peroxidation, protein carbonylation expression, and attenuated protein damage (Martinez-Oliveira et al., 2018).

The Wen-Pi-Tang crude extract (*Rheum officinale* BAILLON, *Panax ginseng* C. A. MEYER, *Aconitum japonicum* THUNBERG, *Zingiber officinale ROSCOE*, *Glycyrrhiza glaba* LINN. var. glandulífera REGEL et HERDER) was administered in rats at 100 or 200 mg/kg/weight/day from 7 to 16-week of age to treat amyotrophic lateral sclerosis (ALS). The treatment preserved the motor function, delayed the onset of symptoms, decreased the rate of neuronal loss in a dose-dependent manner, inhibited immunoreactivity to astrocytes, and reduced the oxidative stress in the spinal cord (considering the 200 mg/kg dose); however, an increase in nNOS expression was observed (Sekiya et al., 2009).

The crude extract of Buyang Huanwu [*Astragalus membranaceus* (Fisch.) Bge. var. *mongholicus* (Bge.) Hsiao; *Angelica sinensis* (Oliv.) Diels; *Paeonia lactiflora* Pall; *Ligusticum chuanxiong*; *Prunus persica* (L.) Batsch; *Carthamus tinctorius* L; *Pheretima aspergillum* (BYHWD)] was prepared and intragastrically administered at 14.8 g/kg/day in rats transplanted or not with embryonic neural tube-derived neural stem cells, which were evaluated after 7, 14, and 28 days of injury in the spinal cord. The treatment induced higher survival and proliferation of neural stem cells in the injured spine, which were also differentiated into astroglia cells and oligodendrocytes. Furthermore, the extract improved myelination, preserved the blood–brain barrier, and enhanced motor and hind limb functions (Zhang et al., 2015).

The crude extract of *Huolingshengji formula* (HLSJ) was prepared with 6 herbs (*Epimedium herb*, *Radix astragali*: *Fructus corni*, *Radix rehmanniae*: *Poria cocos*, *Atractylodes macrocephala Koidz*) considering the respective proportion of 5:6:4:5:3:3. The herbs were smashed and suspended in distilled water at the ratio of 1:10 (w/v). The middle dose of HLSJ (M-HLSJ; 4.5 g/kg·d HLSJ suspended in ddH_2_O) was orally administered for 57 days. The treatment improved the phenotype of the disease (typical movement of the hind limbs), prolonged the lifespan of the animals to 140.67 days, increased the survival rate of motor neurons located in the lumbar spinal cords, and recovered the grip strength of the animals. M-HLSJ decreased gastrocnemius muscle atrophy through the increase of neuromuscular junctions, reduced the expression of apoptotic proteins (Bax by 42%, caspase by 38%, and Cyt c by 31%), as well as increased the expression of the anti-apoptotic protein Bcl2 by 35%. NADH staining and measurement of MDA levels demonstrated cell death decrease and alteration of immune and inflammatory responses through the reduction of the activity of glial cells and inflammation-related proteins (TNF-α, Cox 2, and iNOS; Zhou et al., 2018).

Regarding the compounds obtained from plants, *Acanthopanax senticosus* saponins (ASS) and Gin were extracted from *Acanthopanax senticosus Harms and Ginkgo biloba* tree, respectively. The methanolic extracts were prepared at 50 μg/ml (ASS) and 37.5 μg/ml (Gin), and preincubated with embryonic spinal cord neurons 24 h after their culture and 24 h before hypoxia surgery. The results showed an increased number of fibroblasts, neuroglia, and neurons in the cortical cerebral layer, while LDH release was decreased; however, the HIF-1α levels were increased (Chen et al., 2012).

Proanthocyanidins obtained from *Vitis vinifera L.* fruits and diluted in 0.9% saline solution were administered through intraperitoneal injection in mice at 5 mg/kg or 10 mg/kg between the fifth and tenth day after injury. Forty-eight to 72 h after injury, the treatment promoted locomotive repair by decreasing the iron content in the spinal cord. The reduction in the levels of thiobarbituric acid reactive species (TBARS) indicated a decrease in lipid peroxidation; in addition, the activity of ferroptosis-related molecules (ACSL4 and Alox 15B) was reduced. Both doses increased the activity of erythroid-related factor 2 (Nrf2) and the antioxidant protein glutathione peroxidase 4 (GPx4; Zhou et al., 2020).

The molecule shogaol was isolated from the ground rhizomes of *Zingiber officinale L.*, diluted with Matrigel and dimethyl sulfoxide (DMSO), and administered at different doses (0, 20, 50, and 100 μg/ kg) into the injured spinal cord. Shogaol blocked the PARP expression protein in a dose-dependent manner and consequently decreased cell death, increased the expression of the Bcl2 survival protein, protected against demyelination, and avoided neuron loss into the epicenter area of the spinal cord injury; thus, improvement of hind limb reflex and body weight gain were observed (Kyung et al., 2006).

The Periplocoside E (PSE) molecule was isolated from the stem barks of *Periploca sepium* Bge and administered as an ethanolic extract at 10 mg/kg/day for 16 days after the injury in the spinal cord. The treatment inhibited the severity and duration of clinical paralysis of multiple sclerosis and reduced the infiltration of mononuclear cells and demyelination (Zhu et al., 2006). PSE administration decreased the expression of CD4^+^, CD8^+^, and CD11^+^ mRNA, and inhibited the expression of CCR5 and CXCR3 on T cells present in the lymphoid organs of the rats. The activity of IL-12 and IFN-y inflammatory cytokines was suppressed and thus inhibited T-cell infiltration in the spinal cord. The activity of certain chemokines was down-regulated (CCL2, CCL3, CCL4, CCL5, CXCL9, and CXCL10), which prevented a second influx of T cells into the spinal cord (Zhu et al., 2006; Table 1).

## Discussion

Systematic reviews of animal studies are not often found in the literature; however, they can provide reliable evidence as long as eligibility criteria are established (Hooijmans et al., 2014). Although a great number of *in vivo* and *in vitro* studies on the neuroprotective effects of plants were found, only 14 studies met the eligibility criteria (Figure 1). The crude extracts, compounds, and isolated molecules obtained from plants showed neuroprotective effects against CNS injuries through the increase of antioxidant enzyme activity, modulation of inflammatory response and tissue preservation (Table 1). The selected articles did not discuss which parts of the plants provided the optimal extraction yield of crude extract, compounds, or isolated molecules. Regarding the quality of this review, none of the selected articles showed a high risk of bias in the main domains (Figures 2, 3).

The quality of the evidence found in a systematic review depends on the careful assessment of the risk of bias (Hooijmans et al., 2014). Among the 10 domains used in this review to assess the methodological quality of the studies, only ‘blinding of caregivers and researchers’ and ‘blinding of outcome assessor’ were found unclear in most studies due to the lack of detailing of some conditions and outcome assessments (Figures 2, 3). Moreover, these two domains were not considered critical to determining methodological quality.

The crude extracts can induce different neuroprotective pathways against several injuries such as spinal cord ischemia, spinal cord injury, multiple sclerosis, excitotoxicity injury in the motor cortex, Alzheimer’s disease, and amyotrophic lateral sclerosis (Sekiya et al., 2009; Guimarães-Santos et al., 2012; Badem et al., 2014; Zhang et al., 2015; Lee et al., 2017; Li et al., 2018; Martinez-Oliveira et al., 2018; Huang et al., 2019; Kim et al., 2020). Neuroprotection can be triggered either by the activation of antioxidant enzymes (Martinez-Oliveira et al., 2018) or the downregulation of inflammatory signaling pathways (Guimarães-Santos et al., 2012; Zhang et al., 2015; Lee et al., 2017; Li et al., 2018; Zhou et al., 2018; Huang et al., 2019; Kim et al., 2020) or both (Sekiya et al., 2009). Nevertheless, these events preserve nervous tissue and attenuate the damage caused by injuries in CNS (Sekiya et al., 2009; Guimarães-Santos et al., 2012; Badem et al., 2014; Zhang et al., 2015; Lee et al., 2017; Li et al., 2018; Martinez-Oliveira et al., 2018; Huang et al., 2019; Kim et al., 2020).

The regulation of the antioxidant activity induced by plant crude extracts can occur through the activation of enzymes (SOD, CAT, GSH, and GSH-Px) that decrease the expression of MDA, which is a lipid peroxidation biomarker (Stys, 2004; Martinez-Oliveira et al., 2018). The crude extract of *S. sonchifolius* used to treat spinal cord ischemia, and Wen-Pi-Tang used to treat amyotrophic lateral sclerosis increased the activity of antioxidant enzymes that protect the CNS against the oxygen and nitrogen reactive species due to free radical scavenging, prevent oxidative stress and apoptosis (Sekiya et al., 2009; Badem et al., 2014; Martinez-Oliveira et al., 2018). Although Badem et al. (2014) observed a neuroprotective effect in the histopathological analysis, it must be emphasized that the biochemical evaluation did not indicate any improvement, which may be related to the low percentage of ginkgolides (3.1%) in the *Ginkgo biloba* leaves used to prepare the extract (Luo and Smith, 2004).

**Figure 4:**
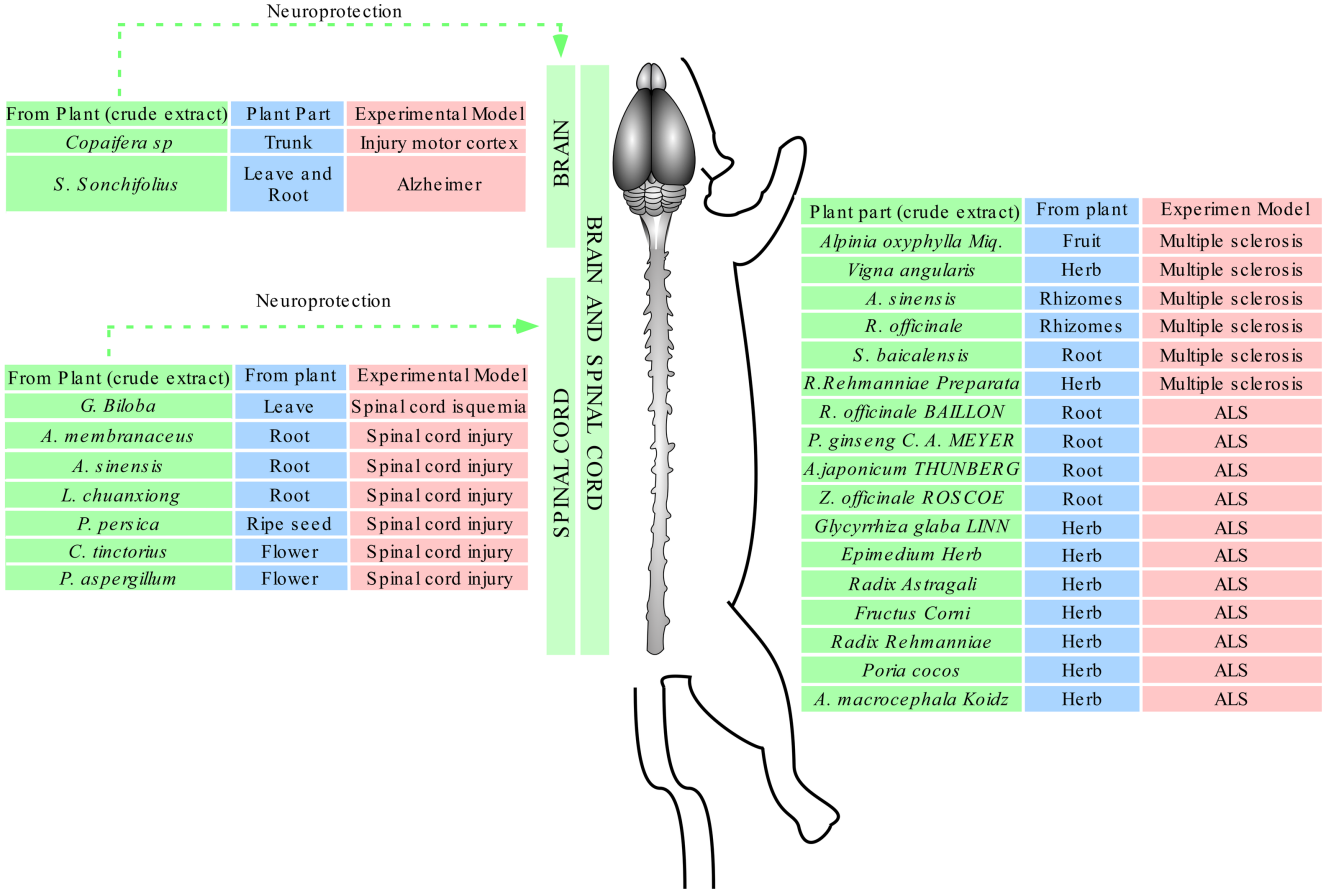
Effects of plant crude extracts against different injuries. Green: plant species; blue: part of the plant used to produce the extract; red; experimental model of injury.

The downregulation of inflammatory signaling pathways such as TRL2 (NF-κB) and MAPKs was observed after treatment with plant crude extracts (Lee et al., 2017; Li et al., 2018; Kim et al., 2020). These events were induced by *Copaifera* oil-resin in excitotoxicity injury in the motor cortex, by *A.oxyphylla Miq.*, *V. angularis*, *C. chinensis FRANCH, S. baicalensis GEORGI*, *R. rehmanniae Preparata* in multiple sclerosis, as well as Wen-Pi-Tang and HLSJ formulas in amyotrophic lateral sclerosis, which deregulated NF-κB and MAPks signaling, inhibited the expression of proinflammatory molecules (Myd88, IRAK4, TRAF6, and COX-2) and consequently activated proinflammatory cytokines (IL-1β, IL-6, IL-8, and TNF-α) and recruited immune T cells (CD3^+^, CD4^+^, and CD8^+^ T cells, CD45 and CD11b^+^) as well of microglia to the injured area (Lee et al., 2017; Li et al., 2018; Huang et al., 2019; Kim et al., 2020). The inhibition of these inflammatory biomarkers decreased cell infiltration, preserved blood–brain barrier integrity, and lead to motor and cognitive improvement (Sekiya et al., 2009; Guimarães-Santos et al., 2012; Zhang et al., 2015; Lee et al., 2017; Li et al., 2018; Zhou et al., 2018; Huang et al., 2019; Kim et al., 2020). However, the mechanisms involved in proinflammatory cytokines expression need to be better elucidated (Sekiya et al., 2009; Guimarães-Santos et al., 2012; Zhang et al., 2015; Lee et al., 2017; Li et al., 2018; Zhou et al., 2018; Huang et al., 2019; Kim et al., 2020; Figure 4).

Treatment of amyotrophic lateral sclerosis with Wen-Pi-Tang extract involved the modulation of two isoforms of nitric oxide synthase: an increase of inducible oxide nitric synthase (iNOS) and a decrease of neuronal oxide nitric synthase (nNOS; Sekiya et al., 2009). The nNOS is responsible to regulates cerebral blood flow and participates in memory formation, while iNOS is related to the product nitric oxide and can cause DNA fragmentation (Cerqueira and Yoshida, 2002). Endothelial oxide nitric synthase (eNOS) is another isoform present in the vascular endothelium, albeit some reports suggest that this enzyme is present in the hippocampus (Wong and Marsden, 1996).

The ASS and Gin compounds produced with saponins and ginkgolides, respectively, found in *Acanthopanax senticosus Harms* and *Ginkgo biloba* and used to treat spinal cord injury (Chen et al., 2012) demonstrated neuroprotective response through inhibition of hypoxia and decrease of LDH release, which lead to higher survival rates of fibroblasts, neuroglial cells, and neurons (Chen et al., 2012). Hypoxic conditions activated a subunit of hypoxia-inducible factor-1 (HIF-1) named HIF-1α, which is considered an oxidative stress marker and acts as a neuroprotective or death transcription factor, depending on the hypoxia severity (He et al., 2021). It has been shown that under moderate hypoxia, the protein may act as a protector by inducing the expression of erythropoietin (EPO-R) and endothelial growth factor (VEGF-R) receptors, which consequently enables oxygen transport and glucose metabolism, as well as stimulates angiogenesis (Freeman and Barone, 2005). However, under severe hypoxia, the protein heterodimerizes with the HIF-1β subunit, translocate to the nucleus, and initiates the transcription of genes whose domains contain a rare hypoxia response element (HRE; Bazan et al., 2002). Under these conditions, the release of reactive oxygen species induces inflammation and cell death by apoptosis (Merelli et al., 2018) Figure 5.

**Figure 5:**
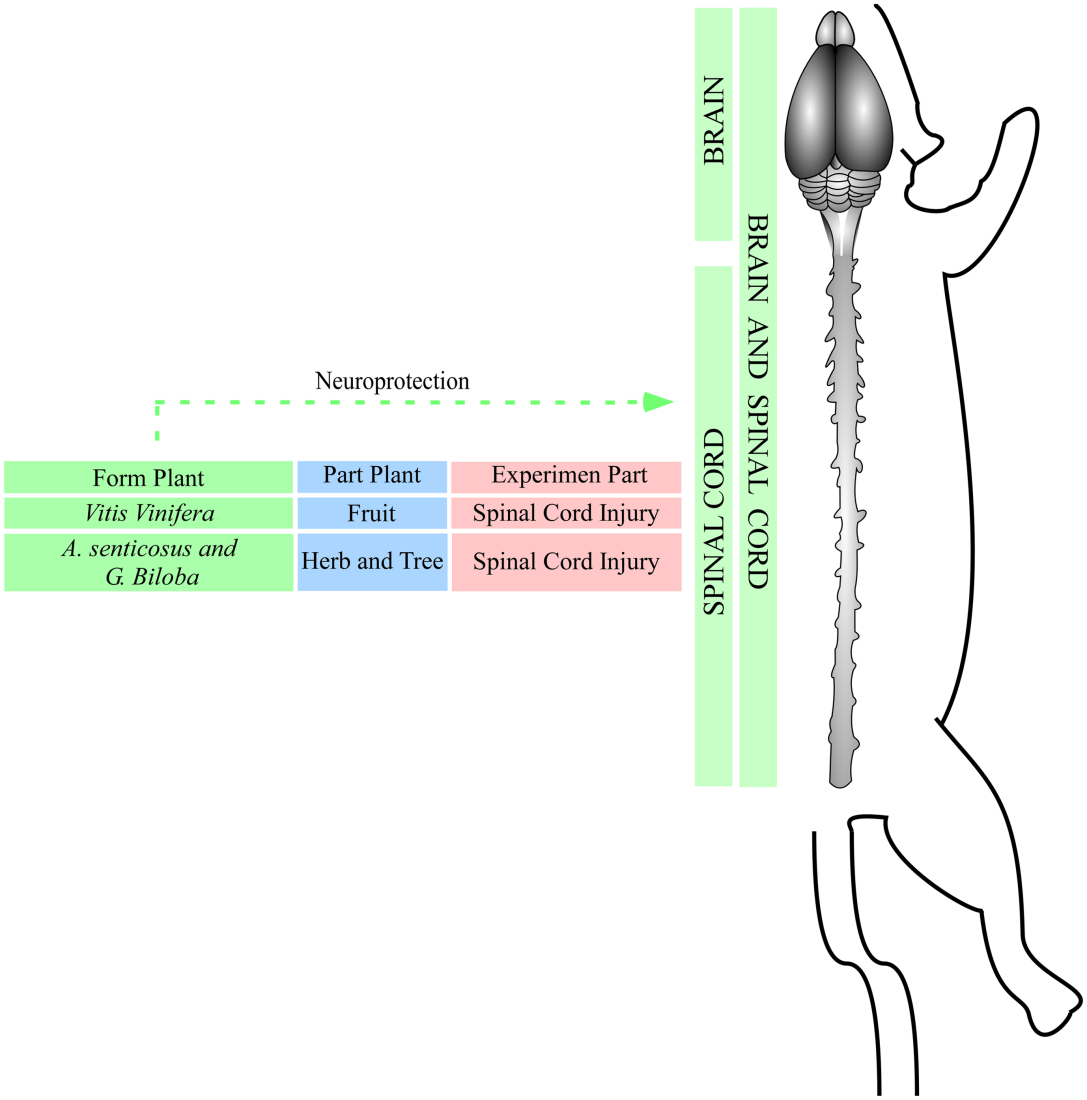
Effects of compounds against injuries. Green: plant species; blue: part of the plan t used to obtain the compounds; red: experimental model of injury.

Ferroptosis is an iron-dependent cell death, in which lethal levels of lipid hydroperoxides are accumulated in the cell membrane (Stoyanovsky et al., 2019). The suppression of these mechanisms occurs through the activation of the transcription factor Nrf2, which is responsible to inhibit some genes related to cell death (Alox 15B and ACSL4); thus, the activation of GSH and GPx4 antioxidant proteins detoxifies membrane lipids (Abdalkader et al., 2018). The proanthocyanidins obtained from *Vitis vinifera L.* fruits and used to treat spinal cord injury showed antioxidant activity by increasing the expression of Nrf2, which inhibited ferroptosis and consequently inhibited the related genes (Zhou et al., 2020).

**Figure 6:**
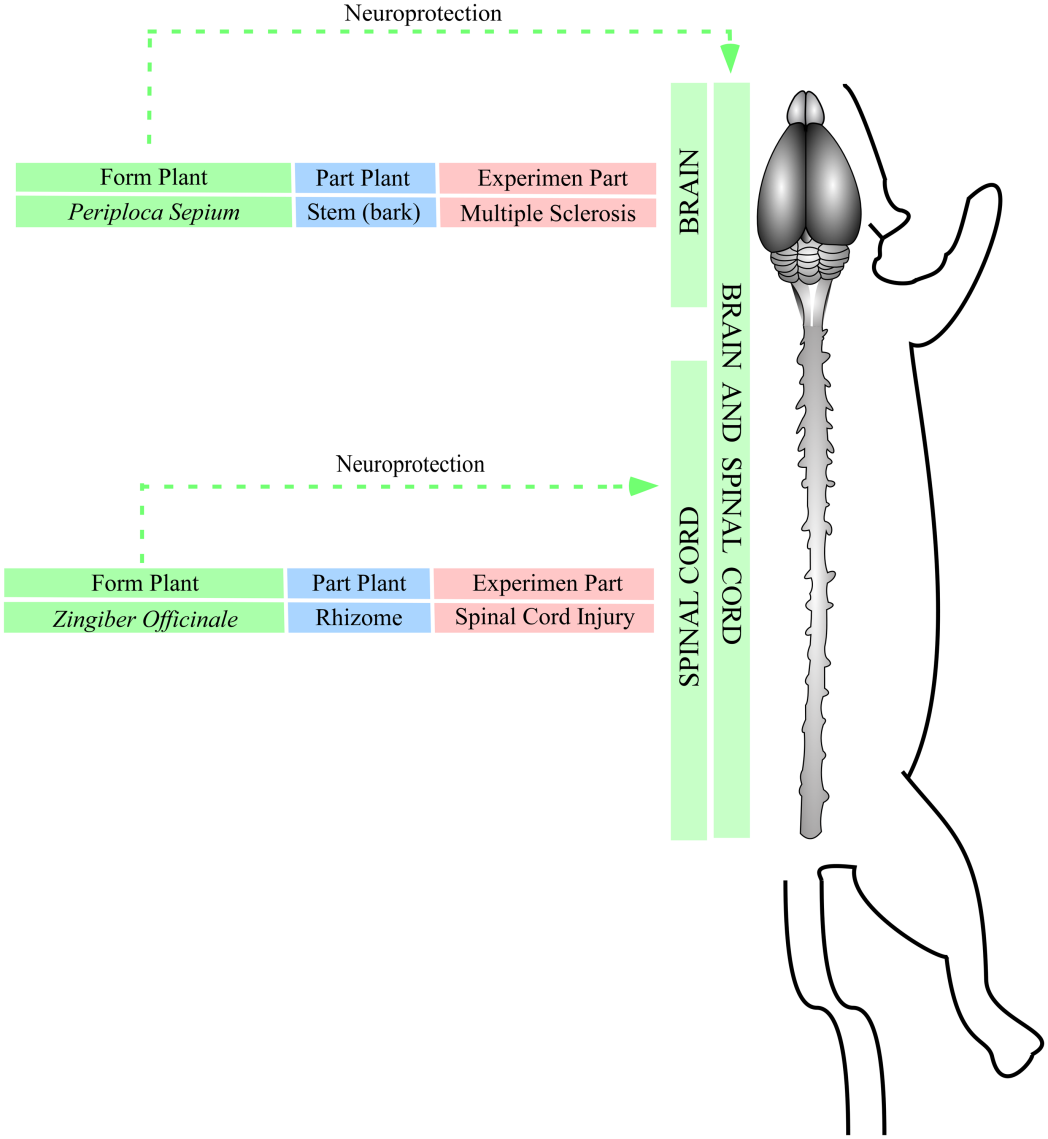
Effects of isolated molecules against injuries. Green: plant species; blue: part of the plan t used to isolate the molecules; red: experimental model of injury.

The molecules shogaol and PSE, which were, respectively, isolated from *Zingiber officinale L.* and *Periploca sepium* Bge, were used to treat spinal cord injury (Kyung et al., 2006) and multiple sclerosis (Zhu et al., 2006) through the activation of anti-apoptotic proteins, hypoxia reduction, tissue preservation, immune response modulation (Figure 6). These events indicate that neuroprotective effects can occur by increasing the activity of anti-apoptotic proteins such as Bcl2, reducing astrogliosis and hypomyelination, and inhibiting apoptotic proteins such as caspase-3 and PARP (Kyung et al., 2006). The suppression of the receptors CCR5 and CXR3 at T lymphocytes inhibits inflammatory cytokines such as IL-12 e IFN-y, which can avoid a second influx of immune cells (astroglia, macrophages, CD4, CD8, and CD11b) and increases tissue survival rate (Glabinski et al., 1995; Zhu et al., 2006; Figure 6).

Therefore, the natural products obtained from different parts of the plants, even when diluted in distilled water, alcohol, or oil-resin, can induce neuroprotective effects against several injuries in CNS, through the modulation of the endogenous antioxidant system, inflammatory response and nervous tissue preservation (Kyung et al., 2006; Zhu et al., 2006; Sekiya et al., 2009; Chen et al., 2012; Guimarães-Santos et al., 2012; Badem et al., 2014; Zhang et al., 2015; Lee et al., 2017; Li et al., 2018; Martinez-Oliveira et al., 2018; Zhou et al., 2018, 2020; Huang et al., 2019; Kim et al., 2020; Table 1).

The extraction and dilution methods did not interfere with the neuroprotective effect provided by natural products from the selected studies. For instance, most of the articles that reproduced the model of spinal cord injury used different diluents (methanol, saline solution). Although the treatment time was different, the administration of a single dose already demonstrated a neuroprotective effect, which seems related to the concentration of the natural product. Although the optimal therapeutic window could not be determined, those treatments initiated soon after the injuries showed the best outcomes (Guimarães-Santos et al., 2012; Kim et al., 2020).The doses of the natural products also varied (Kyung et al., 2006; Zhu et al., 2006; Sekiya et al., 2009; Chen et al., 2012; Guimarães-Santos et al., 2012; Badem et al., 2014; Zhang et al., 2015; Lee et al., 2017; Li et al., 2018; Martinez-Oliveira et al., 2018; Zhou et al., 2018, 2020; Huang et al., 2019; Kim et al., 2020); however, only the extract of *Gingko biloba* leaves at 100 mg/kg did not result in biochemical alterations after treatment (Badem et al., 2014). Furthermore, age and gender seem not determinant factors of neuroprotective effects since this review observed that male and female animals at different ages, as well as cell cultures, benefit from treatments with crude extracts, compounds, and isolated molecules (Kyung et al., 2006; Zhu et al., 2006; Sekiya et al., 2009; Chen et al., 2012; Guimarães-Santos et al., 2012; Badem et al., 2014; Zhang et al., 2015; Lee et al., 2017; Li et al., 2018; Martinez-Oliveira et al., 2018; Zhou et al., 2018, 2020; Huang et al., 2019; Kim et al., 2020). Therefore, the concentration of the natural product figures as a key factor for the treatment success (Kyung et al., 2006; Zhu et al., 2006; Sekiya et al., 2009; Chen et al., 2012; Guimarães-Santos et al., 2012; Badem et al., 2014; Zhang et al., 2015; Lee et al., 2017; Li et al., 2018; Martinez-Oliveira et al., 2018; Zhou et al., 2018, 2020; Huang et al., 2019; Kim et al., 2020). Although there is a great worldwide diversity of plants with potential neuroprotective effects, unfortunately, only studies with a few plant species were found in this systematic review.

## Conclusion

Natural products obtained from plants in the forms of crude extracts, compounds, or isolated molecules showed promising neuroprotective effects against several CNS injuries in both the brain and spinal cord, regardless of gender and age, through the modulation of inflammatory activity and oxidative biochemistry, tissue preservation, recovery of motor and cognitive activity. Only a single dose of natural product can induce neuroprotective effects; however, the concentration and the immediate start are key factors for the treatment success. In addition, neuroprotective effects are not restricted to a single plant family, and the most efficient part of each plant is not determined. Further studies are needed to determine which parts of the plants better induce neuroprotective effects, to determine the optimal therapeutic window, and to standardize the experimental model.

## Data availability statement

The original contributions presented in the study are included in the article/Supplementary material, further inquiries can be directed to the corresponding author.

## Author contributions

All authors had full access to complete data and took responsibility for the integrity of the information, text, and analysis accuracy. MM and GM contributed equally to this work. CB: study concept and design, critical revision for important intellectual content, funding obtained, and supervision. GM, MM, MA, and MG: data acquisition. ES and TL: analysed the data and prepared the figures and table. KO and AH: contributed to critical revision for important intellectual content. GM and MM: manuscript drafting. All authors contributed to the article and approved the submitted version.

## Funding

This study was supported by the National Council for Scientific and Technological Development – CNPq (grants no. 310054/2018-4, 447835/2014-9, 483404/2013-6, 444967/2020-6, and 444982/2020-5) and the Brazilian Agency for Support and Evaluation of Graduate Education – CAPES (grant no. PROCAD 21/2018).

## Conflict of interest

The authors declare that the research was conducted in the absence of any commercial or financial relationships that could be construed as a potential conflict of interest.

## Publisher’s note

All claims expressed in this article are solely those of the authors and do not necessarily represent those of their affiliated organizations, or those of the publisher, the editors and the reviewers. Any product that may be evaluated in this article, or claim that may be made by its manufacturer, is not guaranteed or endorsed by the publisher.

## Supplementary material

The Supplementary material for this article can be found online at: https://www.frontiersin.org/articles/10.3389/fnins.2023.1249685/full#supplementary-material

## Footnotes

^1^ doi.org/10.17605/OSF.IO/ZGMQJ

^2^ https://rayyan.ai/

## Glossary

ASS: Acanthopanax senticosus
AO-1: Alpinia oxyphylla
ALS: Amyotrophic lateral sclerosis
BBB: Blood–brain barrier
BYHWD: Buyang Huanwu
CNS: Central nervous system
COR: Copaifera reticulata
CAT: Catalase
DMSO: Dimethyl sulfoxide
eNOS: Endothelial nitric oxide synthase
Gin: Ginkgolides
GSH: Glutathione
GSH-Px: Glutathione peroxidase
HIF-1α: Hypoxia-inducible factor-1-α
HLSJ: Huolingshengji
HO-1: Heme oxygenase-1
iNOS: Nitric oxide interstitial synthase
IL-12: Interleukin-12
IFN-γ: Interferon- γ
LDH: Lactate dehydrogenase
MDA: Malonic dialdehyde
MAPKs: Map kinases
NADH: Nicotinamide adenine dinucleotide
NF-kβ: Fator nuclear kβ
NO: Nitric oxide
nNOS: Nitric oxide neuronal synthase
NSCs: Embryonic neural tube cells
OAA: Oleanolic acid acetate
ONOO−: peroxynitrite
PACs: Proanthocyanidins
PSE: Periplocoside E
ROS: Reactive oxygen species
RR: Radix rehmannia
SCI: Spinal cord injury
SHSST: Samhwangsasim-tang
SOD: Superoxide dismutase
TBARS: Thiobarbituric acid reactive species
TNF-α: Tumor necrosis factor α
