# Supplementary material for: Neuroprotective effects of crude extracts, compounds, and isolated molecules obtained from plants in the central nervous system injuries: a systematic review
Source: Front Neurosci. 2023 Sep 12;17:1249685. doi: 10.3389/fnins.2023.1249685 (PMC10520969; doi:10.3389/fnins.2023.1249685)
Supplement: Supplementary file 1 [file Table_1.DOCX]

Title: Neuroprotective effects of plant extract and isolated compounds on neurodegenerative diseases: a systematic review

1. PICO Search Strategy

PICo: “Do plants have a neuroprotective effect?”

|  | **Key-words** | **MESH** | **ENTRY TERMS** |
| --- | --- | --- | --- |
| **P** | Animals | Animals | Animalia  Animal  Metazoa |
| **I** | Medicinal plants | Medicinal plants | Medicinal Plant  Plant, Medicinal  Medicinal Plants  Medicinal Herbs  Herb, Medicinal  Medicinal Herb  Herbs, Medicinal  Pharmaceutical Plants  Pharmaceutical Plant  Plant, Pharmaceutical  Plants,Pharmaceutical  Healing Plants  Healing Plant  Plant, Healing  Plants, Healing |
|  | Fruit | Fruit | Fruits  Plant Capsule  Capsule, Plant  Capsules, Plant  Plant Capsules  Plant Aril  Aril, Plant  Arils, Plant  Plant Arils  Berries  Berry  Legume Pod  Legume Pods  Pod, Legume  Pods, Legume |
| **C** | Control | Control | ----------------------------------- |
| **O** | Neuroprotection | Neuroprotection | Neural Protection  Protection, Neural  Neuron Protection  Protection, Neuron  Neuronal Protection  Protection, Neuronal |
|  | Central Nervous System | Central Nervous System | Central Nervous Systems  Nervous System, Central  Nervous Systems, Central  Systems, Central Nervous  Cerebrospinal Axis  Axi, Cerebrospinal  Axis, Cerebrospinal  Cerebrospinal Axi |
|  | Spinal Cord | Spinal Cord | Cord, Spinal  Cords, Spinal  Spinal Cords  Medulla Spinalis  Medulla Spinali  Spinali, Medulla  Spinalis, Medulla  Myelon  Myelons  Coccygeal Cord  Coccygeal Cords  Cord, Coccygeal  Cords, Coccygeal  Conus Medullaris  Conus Medullari  Medullari, Conus  Medullaris, Conus  Conus Terminalis  Conus Terminali  Terminali, Conus  Terminalis, Conus  Thoracic Cord  Cord, Thoracic  Cords, Thoracic  Thoracic Cords  Lumbar Cord  Cord, Lumbar  Cords, Lumbar  Lumbar Cords  Sacral Cord  Cord, Sacral  Cords, Sacral  Sacral Cords |

| **Database** | **Total articles** |
| --- | --- |
| PubMed | 5,427 |
| Scopus | 4 |
| Science Direct | 90 |
| Total | 5,521 |
| Web sites | 2 |
| **Duplicates removed** | 32 |
| **Included articles** | 14 |

Search strategy in PubMed

(Animalia[Title/Abstract]) OR (Animal[Title/Abstract])) OR (Metazoa[Title/Abstract])) AND (Medicinal Plant[Title/Abstract])) OR (Plant, Medicinal[Title/Abstract])) OR (Medicinal Plants[Title/Abstract])) OR (Medicinal Herbs[Title/Abstract])) OR (Herb, Medicinal[Title/Abstract])) OR (Medicinal Herb[Title/Abstract])) OR (Herbs, Medicinal[Title/Abstract])) OR (Pharmaceutical Plants[Title/Abstract])) OR (Pharmaceutical Plant[Title/Abstract])) OR (Plant, Pharmaceutical[Title/Abstract])) OR (Plants, Pharmaceutical[Title/Abstract])) OR (Healing Plants[Title/Abstract])) OR (Healing Plant[Title/Abstract])) OR (Plant, Healing[Title/Abstract])) OR (Plants, Healing[Title/Abstract])) AND (Fruit [Title/Abstract])) OR (Fruits [Title/Abstract])) OR (Plant Capsule [Title/Abstract])) OR (Capsule, Plant [Title/Abstract])) OR (Capsules, Plant [Title/Abstract])) OR (Plant Capsules [Title/Abstract])) OR (Plant Aril [Title/Abstract])) OR (Aril, Plant [Title/Abstract])) OR (Arils, Plant [Title/Abstract])) OR (Plant Arils [Title/Abstract])) OR (Berries[Title/Abstract])) OR (Berry [Title/Abstract])) OR (Legume Pod [Title/Abstract])) OR (Legume Pods [Title/Abstract])) OR (Pod, Legume [Title/Abstract])) OR (Pods, Legume [Title/Abstract])) AND (Neural Protection[Title/Abstract])) OR (Protection, Neural[Title/Abstract])) OR (Neuron Protection[Title/Abstract])) OR (Protection, Neuron[Title/Abstract])) OR (Neuronal Protection[Title/Abstract])) OR (Protection, Neuronal[Title/Abstract])) AND (Central Nervous Systems[Title/Abstract])) OR (Nervous System, Central[Title/Abstract])) OR (Nervous Systems, Central[Title/Abstract])) OR (Systems, Central Nervous[Title/Abstract])) OR (Cerebrospinal Axis[Title/Abstract])) OR (Axi, Cerebrospinal[Title/Abstract])) OR (Axis, Cerebrospinal[Title/Abstract])) OR (Cerebrospinal Axi[Title/Abstract])) AND (Brain[Title/Abstract]) OR Brain[Title/Abstract]) OR Encephalon[Title/Abstract]) AND (Cord, Spinal[Title/Abstract])) OR (Cords, Spinal[Title/Abstract])) OR (Spinal Cords[Title/Abstract])) OR (Medulla Spinalis[Title/Abstract])) OR (Medulla Spinali[Title/Abstract])) OR (Spinali, Medulla[Title/Abstract])) OR (Spinalis, Medulla[Title/Abstract])) OR (Coccygeal Cord[Title/Abstract])) OR (Coccygeal Cords[Title/Abstract])) OR (Cord, Coccygeal[Title/Abstract])) OR (Cords, Coccygeal[Title/Abstract])) OR (Cords, Coccygeal[Title/Abstract])) OR (Conus Medullaris[Title/Abstract])) OR (Conus Medullari[Title/Abstract])) OR (Medullari, Conus[Title/Abstract])) OR (Medullaris, Conus[Title/Abstract])) OR (Conus Terminalis[Title/Abstract])) OR (Conus Terminali[Title/Abstract])) OR (Terminali, Conus[Title/Abstract])) OR (Terminalis, Conus[Title/Abstract])) OR (Thoracic Cord[Title/Abstract])) OR (Cord, Thoracic[Title/Abstract])) OR (Cords, Thoracic[Title/Abstract])) OR (Thoracic Cords[Title/Abstract])) OR (Lumbar Cord[Title/Abstract])) OR (Cord, Lumbar[Title/Abstract])) OR (Cords, Lumbar[Title/Abstract])) OR (Lumbar Cords[Title/Abstract])) OR (Sacral Cord[Title/Abstract])) OR (Cord, Sacral[Title/Abstract])) OR (Cords, Sacral[Title/Abstract])) OR (Sacral Cords[Title/Abstract])

Search strategy in Scopus

TITLE-ABS-KEY ( Animals ) OR TITLE-ABS-KEY ( Animalia ) OR TITLE-ABS-KEY ( Animal ) OR TITLE-ABS-KEY ( Metazoa )

AND

TITLE-ABS-KEY ( “Medicinal Plant” ) OR TITLE-ABS-KEY ( “Plant, Medicinal” ) OR TITLE-ABS-KEY ( “Medicinal Plants” ) OR TITLE-ABS-KEY ( “Medicinal Herbs”) OR TITLE-ABS-KEY ( “Herb, Medicinal” ) OR TITLE-ABS-KEY ( “Medicinal Herb” ) OR TITLE-ABS-KEY ( “Herbs, Medicinal” ) OR TITLE-ABS-KEY ( “Pharmaceutical Plants” ) OR TITLE-ABS-KEY ( “Pharmaceutical Plant” ) OR TITLE-ABS-KEY ( “Plant, Pharmaceutical” ) OR TITLE-ABS-KEY ( “Plants, Pharmaceutical” ) OR TITLE-ABS-KEY ( “Healing Plants” ) OR TITLE-ABS-KEY ( “Healing Plant” ) OR TITLE-ABS-KEY ( “Plant, Healing” ) OR TITLE-ABS-KEY ( “Plants, Healing” )

AND

TITLE-ABS-KEY ( Fruit ) OR TITLE-ABS-KEY ( Fruits ) OR TITLE-ABS-KEY ( “Plant Capsule” ) OR TITLE-ABS-KEY ( “Capsule, Plant” ) OR TITLE-ABS-KEY ( “Capsules, Plant” ) OR TITLE-ABS-KEY ( “Plant Capsules” ) OR TITLE-ABS-KEY ( “Plant Aril” ) OR TITLE-ABS-KEY ( “Aril, Plant” ) OR TITLE-ABS-KEY ( “Arils, Plant” ) OR TITLE-ABS-KEY ( “Plant Arils” ) OR TITLE-ABS-KEY ( Berries) OR TITLE-ABS-KEY ( Berry ) OR TITLE-ABS-KEY ( “Legume Pod” ) OR TITLE-ABS-KEY ( “Legume Pods” ) OR TITLE-ABS-KEY ( “Pod, Legume” ) OR TITLE-ABS-KEY ( “Pods, Legume” )

AND

TITLE-ABS-KEY ( “Neural Protection” ) OR TITLE-ABS-KEY ( “Protection, Neural” ) OR TITLE-ABS-KEY ( “Neuron Protection” ) OR ( “Protection, Neuron” ) OR ( “Neuronal Protection” ) OR TITLE-ABS-KEY ( “Protection, Neuronal” )

AND

TITLE-ABS-KEY ( “Central Nervous System” ) OR TITLE-ABS-KEY ( “Central Nervous Systems” ) OR TITLE-ABS-KEY ( “Nervous System, Central” ) OR TITLE-ABS-KEY ( “Nervous Systems Central” ) OR TITLE-ABS-KEY ( “Systems Central Nervous” ) OR TITLE-ABS-KEY ( “Cerebrospinal Axis” ) OR TITLE-ABS-KEY ( “Axi, Cerebrospinal” ) OR TITLE-ABS-KEY ( “Axis Cerebrospinal” ) OR TITLE-ABS-KEY ( “Cerebrospinal Axi” ) OR TITLE-ABS-KEY ( “Spinal Cord” ) OR TITLE-ABS-KEY ( “Cord Spinal” ) OR TITLE-ABS-KEY ( “Cords Spinal” ) OR TITLE-ABS-KEY ( “Spinal Cords” ) OR TITLE-ABS-KEY ( “Medulla Spinalis” ) OR TITLE-ABS-KEY ( “Medulla Spinali” ) OR TITLE-ABS-KEY ( “Spinali, Medulla” ) OR TITLE-ABS-KEY ( “Spinalis Medulla” ) OR TITLE-ABS-KEY ( Myelon ) OR TITLE-ABS-KEY ( Myelons ) OR TITLE-ABS-KEY ( “Coccygeal Cord” ) OR TITLE-ABS-KEY ( “Coccygeal Cords” ) OR TITLE-ABS-KEY ( “Cord, Coccygeal” ) OR TITLE-ABS-KEY ( “Cords, Coccygeal” ) OR TITLE-ABS-KEY ( “Conus Medullaris” ) OR TITLE-ABS-KEY ( “Conus Medullari” ) OR TITLE-ABS-KEY ( “Medullari, Conus” ) OR TITLE-ABS-KEY ( “Medullaris, Conus” ) OR TITLE-ABS-KEY ( “Conus Terminalis” ) OR TITLE-ABS-KEY ( “Conus Terminali” ) OR TITLE-ABS-KEY ( “Terminali, Conus” ) OR TITLE-ABS-KEY ( “Terminalis, Conus” ) OR TITLE-ABS-KEY ( “Thoracic Cord” ) OR TITLE-ABS-KEY ( “Cord, Thoracic” ) OR TITLE-ABS-KEY ( “Cords, Thoracic” ) OR TITLE-ABS-KEY ( “Thoracic Cords” ) OR TITLE-ABS-KEY ( “Lumbar Cord” ) OR TITLE-ABS-KEY ( “Cord, Lumbar” ) OR TITLE-ABS-KEY ( “Cords, Lumbar” ) OR TITLE-ABS-KEY ( “Lumbar Cords” ) OR TITLE-ABS-KEY ( “Sacral Cord” ) OR TITLE-ABS-KEY ( “Cord, Sacral” ) OR TITLE-ABS-KEY ( “Cords, Sacral” ) OR TITLE-ABS-KEY ( “Sacral Cords” )

Search strategy in Science Direct

Animals + Medicinal Plant + Fruit + Neuroprotection+ Central nervous system + Spinal cord
